# Transjugular Patent Ductus Arteriosus Occlusion in Seven Dogs Using the Amplatzer Vascular Plug II

**DOI:** 10.3390/vetsci9080431

**Published:** 2022-08-14

**Authors:** Mara Bagardi, Oriol Domenech, Tommaso Vezzosi, Federica Marchesotti, Martina Bini, Valentina Patata, Marta Croce, Valentina Valenti, Luigi Venco

**Affiliations:** 1 Cardiology Unit, Anicura Clinica Veterinaria Orobica, Azzano San Paolo, 24052 Bergamo, Italy; 2 Cardiology Unit, Anicura Istituto Veterinario Novara, Granozzo con Monticello, 28060 Novara, Italy; 3 Department of Veterinary Sciences, University of Pisa, 56122 Pisa, Italy; 4 Cardiology Unit, Ospedale Veterinario Città di Pavia, 27100 Pavia, Italy

**Keywords:** jugular vein access, transcatheter closure, Amplatz canine ductal occluder, congenital heart disease, cardiology, canine

## Abstract

**Simple Summary:**

This study outlines the authors’ experience using the Amplatzer Vascular Plug II device for occlusion of patent ductus arteriosus (PDA) in dogs through a right transjugular approach, never before described in veterinary medicine for this purpose. The obtained results demonstrate that the use of the Amplatzer Vascular Plug II via a transvenous right jugular approach is a feasible and effective method, even in small-sized patients. The authors’ experience is that the transvenous approach is a safe and effective method for PDA occlusion in dogs. Furthermore, the right jugular approach provides easier and faster vascular access than the femoral one. The authors affirm that this approach, together with the properties of the Amplatzer Vascular Plug II and its wide size selection, potentially provide a novel method that can complement the current available methods for transcatheter PDA occlusion.

**Abstract:**

Although vascular plugs for the closure of patent ductus arteriosus (PDA) have been validated in dogs, studies are lacking on its use as a first-choice device with a transjugular approach. The present case series describes the transvenous right jugular embolization of PDA using an Amplatzer Vascular Plug II in seven dogs of different ages, breeds, and body weights. Complete closure of the PDA was demonstrated in all cases. All dogs showed significant hemodynamic reduction of pulmonary overcirculation and left heart size after the procedure and at following echocardiographic check-ups. Transjugular PDA occlusion using an Amplatzer Vascular Plug II can thus be considered as a safe alternative to the arterial or venous femoral approach using an Amplatzer canine ductal occluder (ACDO), particularly in puppies with small femoral vessels.

## 1. Introduction

Epidemiological studies on congenital heart diseases in dogs have been conducted globally since the early 1960s [1,2,3,4,5,6,7,8,9,10,11] In almost all studies, the most common congenital heart diseases observed were patent ductus arteriosus (PDA), pulmonic stenosis, and subaortic stenosis [1,2,3,4,5,6,7,8,12]. PDA can occur in any breed; however, small or medium breeds appear to be overrepresented [13,14]. PDA is also more often seen in females than in males [14].

Left-to-right shunting PDA can lead to pathological pulmonary overcirculation and left-sided volume overload, potentially resulting in congestive heart failure [2,15,16]. If PDA occlusion is not performed, 64% of affected dogs may die within one year [15].

Treatment of PDA involves permanent attenuation of the ductal flow, either through surgical ligation or minimally invasive transcatheter occlusion with devices such as the Amplatz canine ductal occluder (ACDO), vascular plugs, or embolization coils released via an arterial or venous approach [17,18,19,20,21]. Among the minimally invasive treatment approaches, transcatheter occlusion with ACDO devices has been reported as the treatment of choice for dogs with left-to-right shunting PDA [17,19,21]. This provides a minimally invasive controlled catheter-based delivery system, easy deployment, low complication rate, a broad range of sizes of the ADCO device and effective closing of a wide range of patients and PDA dimensions [18,21]. However, the ACDO delivery system size and the need to use this device following a transarterial approach may limit its use in small dogs, especially when presenting large-sized PDAs [21].

Using Amplatzer vascular plugs II (AVP II) for interventional closure of PDA through a transvenous approach has recently been reported [22,23]. AVP II is a self-expandable occlusion device made of nitinol wire mesh with lateral retention discs and a central component with the same diameter in a symmetric cylindrical shape. Due to its symmetrical shape, this device can also be used through a transvenous vascular approach using relatively small catheters [22,23].

Placing an introducer vascular access sheath in the femoral vein is safe and effective for PDA occlusion using the AVP II [22,23]. Similarly, as with the arterial vascular access, transvenous vascular access through the femoral vein can be performed either by a surgical cut-down or percutaneous puncture of the femoral vein using the Seldinger technique [24]. The removal of the introducer vascular sheath from the percutaneous femoral vein access may rarely cause a significant hemorrhage and hematoma. This could be managed with compression or prevented by a surgical cut-down with subsequent femoral vein ligation. On the other hand, surgical cut-down of the femoral vein can prolong the procedure time and can be technically difficult in small dogs in comparison to transjugular venous access [25,26].

Right jugular venotomy in human medicine is described as an easier and faster vascular venous access via either the cut-down technique or the percutaneous Seldinger technique compared with the transfemoral approach, and it enables adequate compression to be applied to decrease the risk of hemorrhage and complications [27]. Furthermore, the British Cardiac Society recommends that the right internal jugular vein is the best route in most human patients, particularly during procedures conducted by inexperienced operators. This is because it provides the most direct route to the right ventricle, a high success rate, a shorter procedure duration, and few complications [28,29].

To the best of our knowledge, there are no studies that describe the transjugular occlusion of the PDA with AVP II in dogs and no direct comparisons of outcomes between AVP II implantation with the femoral vein approach and the jugular vein approach have been reported. The aim of this case series was thus to describe a new venous approach through the right jugular vein for PDA closure with AVP II in dogs. This study did not intend to compare the results obtained by transjugular occlusion with AVP II with a control group treated with transcatheter occlusion by ACDO devices.

## 2. Materials and Methods

**Figure 1:**
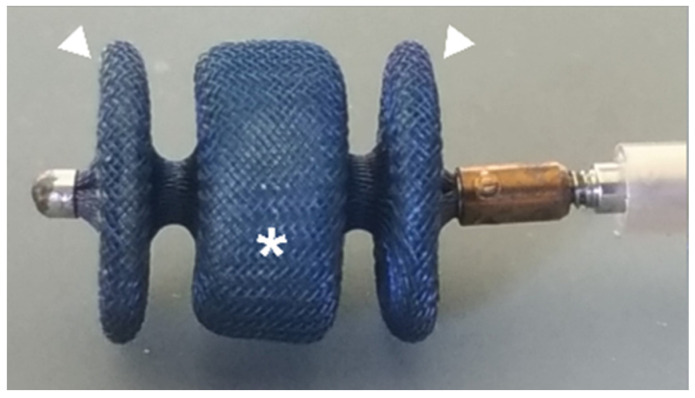
Amplatzer vascular plug II with two lateral retention discs (arrowhead) and central component (asterisk): self-expanding device made from a nitinol wire mesh. The device is attached by a microscrew to a 135 cm nitinol delivery wire in a hoop dispenser and preloaded in a loader.

The procedures described in this study were performed following veterinary good clinical practice guidelines and written informed consent was obtained from the owners to perform the PDA closure. Data regarding dogs that underwent a minimally invasive PDA occlusion with the AVP II (Figure 1) via the transjugular approach were retrospectively reviewed.

The left-to-right shunting PDA was suspected by auscultation of a characteristic continuous murmur at the left axillary area and confirmed by transthoracic echocardiography. All cases also underwent complete hematobiochemical analysis, thoracic radiographs, and electrocardiography. The PDA was visualized from the right parasternal short axis view and from the left parasternal cranial views. Maximal ductal measurements, including the minimal ductal diameter (MDD) and the ampulla diameter (AD), were obtained from the echocardiographic view that provided the highest echocardiographic quality. Measurements were performed by an experienced operator in veterinary interventional procedures (L.V.) and by a board-certified cardiologist (O.D.). The MDD was measured at the level of the ductal opening into the pulmonary artery and the AD was measured perpendicular to the ductal wall at the midway through its long axis. The dimension of the occlusion device was based on the type and size of the duct which was at least twice as large as the minimal ductal diameter and at least 20% larger than the maximal echocardiographic AD measurement [22].

**Figure 2:**
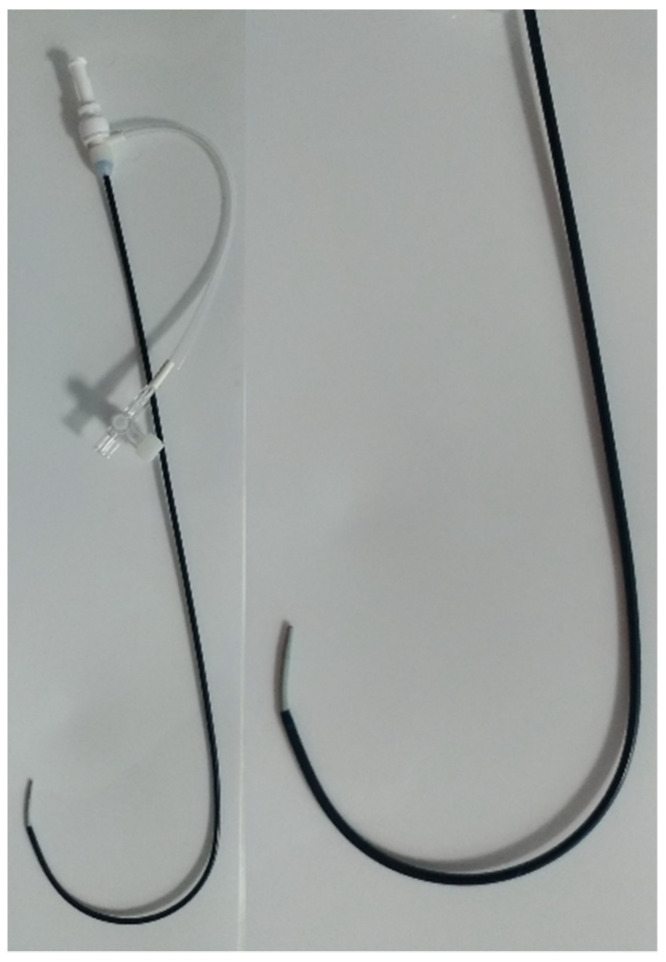
Cook Medical Flexor Balkin Guiding Sheath (5.5 Fr) with Check-Flo Valve and hydrophilic coating. On the right, magnification of the 180° curve guiding sheath.

Premedication included methadone (0.2 mg/kg, intramuscularly [IM]). Anesthesia was induced with fentanyl (3 mcg/kg intravenously [IV]), midazolam (0.2 mg/kg IV) and propofol (3 mg/kg IV). An appropriate plane of anesthesia was maintained, with isoflurane and oxygen delivered via a precision vaporizer and a non-rebreathing circuit. For the procedure, all dogs were placed in left lateral recumbency on the fluoroscopy table. The right jugular vein was surgically isolated by the cut down technique and a 7 or an 8 Fr introducer vascular sheath (PINNACLE^®^ R/O II HIFLO Introducer Sheath 7 Fr × 4 cm × 0.038″ or PINNACLE^®^ PERIPHERAL Introducer Sheath 7 Fr/8 Fr × 10 cm × 0.035″ Terumo Interventional Systems, Tokyo, Japan; Super Sheath™ Introducer Sheath 8 Fr/9 Fr × 11 cm × 0.038″, distributed by Boston Scientific Corporation, Natick, MA, USA, manufactured by Togo Medikit Co., Ltd., Hyūga, Tokyo, Japan) was placed into the right jugular vein. A 4 Fr Berenstein guiding catheter (distributed by Infiniti Medical, Huddersfield, UK and manufactured by Abbott Medical, Plymouth, MN, USA) or 5 Fr multipurpose catheter (Beacon Tip 5 Fr × 0.038″ × 100 cm, Cook Medical Inc, Bloomington, IN, USA) was then inserted into the jugular vein along with a preplaced straight tip soft guidewire (0.035″ Terumo Glidewire, 0.035″ × 150 cm, Straight). It was passed through the right ventricle, the pulmonary artery and the PDA in a retrograde manner until the tip of the guiding catheter or multipurpose catheter was located in the descending aorta under fluoroscopy guidance. After removing the guidewire, an angiographic study using iohexol was performed showing the location and the morphology of the PDA. Following angiography, a Cook Rosen PTFE Curved Wire Guide 0.035″ × 145 cm was advanced into the descending aorta and the Berenstein guiding catheter or multipurpose catheter was removed. Based on the chosen AVP II size, a 5.5 Fr to 6 Fr × 40 cm guiding sheath (Flexor Balkin, Cook Medical Inc, Bloomington, IN, USA) was advanced over the guidewire into the descending aorta (Figure 2) acting as delivering catheter, and the guidewire was subsequently removed.

The 180° curve of the Balkin guiding sheath enables it to easily pass through the right ventricle in order to reach the pulmonary artery, the PDA and the descending aorta.

**Figure 3:**
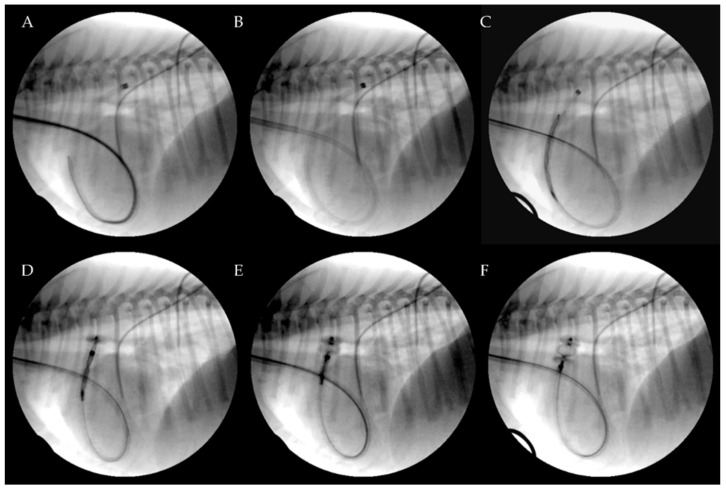
Transjugular patent ductus arteriosus occlusion using the Amplatzer Vascular Plug II. The PDA was crossed retrograde using a straight soft guidewire advanced into the descending aorta through the right ventricle and the pulmonary artery on a 5 Fr multipurpose catheter. The soft guidewire was replaced by a standard Cook Rosen PTFE curved guidewire used to position the tip of the Flexor Balkin guiding sheath into the aorta and was then removed (**A**). The Flexor Balkin guiding sheath is in correct position (**B**). The device was introduced into the Balkin guiding sheath (**C**). The Amplatzer Vascular Plug II was advanced (**C**), partially (**D**) and fully extruded (**E**) and occluding the PDA (**F**).

The device was introduced into the Balkin guiding sheath through the Check-Flo Valve and then advanced carefully until the distal disc was expanded into the descending aorta near the ductus. The partially-deployed AVP II, the attached delivery cable and the guiding sheath were gently pulled back simultaneously, until the distal disc engaged the aortic ostium of the PDA. The guiding sheath then was retracted while slightly pushing the delivery wire, allowing the central component of the device to expand into the PDA ampulla (Figure 3).

**Figure 4:**
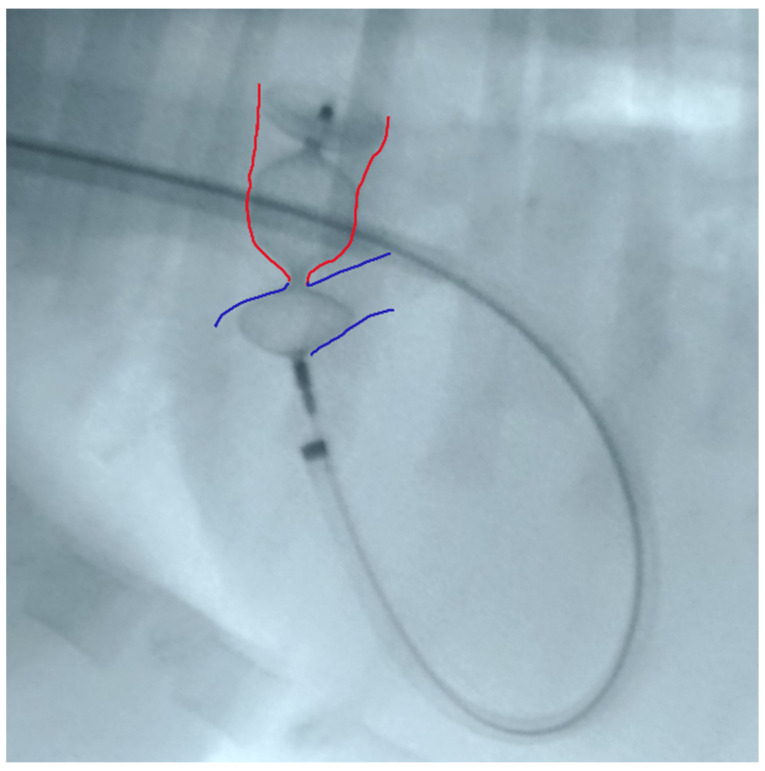
Device deployed in the main pulmonary artery with slight distension of the proximal disc. Red: ductal ampulla; Blue: pulmonary artery.

The proximal disk was then deployed in the main pulmonary artery with a firm but not excessive traction that was sufficient to create a slight distension of the proximal disc. This guarantees its position in the pulmonary artery, while the lack of waist in the device central component indicates that it is totally positioned in the ampulla and is not engaged in the ostium (Figure 4).

**Figure 5:**
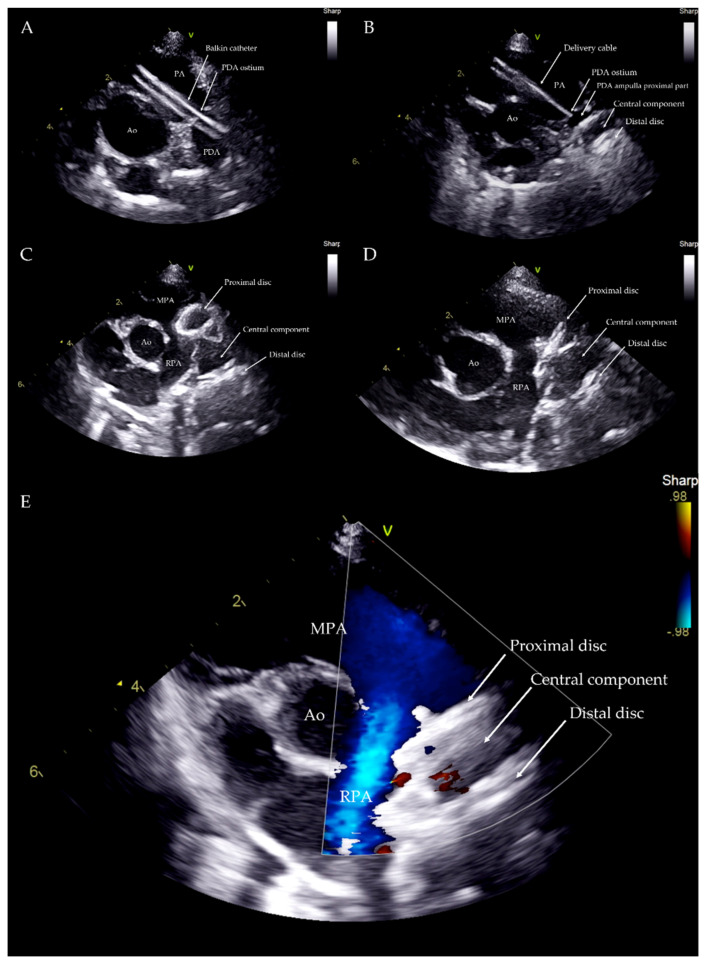
Transthoracic echocardiography from left parasternal cranial view during the procedure. Balkin catheter through the PDA (**A**). Distal disc and central component within the PDA ampulla (**B**). Distal disc and central component within the PDA ampulla and proximal disc within the PA just before the device deployment (**C**). Device released within the PDA structure (**D**). Device within the PDA with no residual flow (**E**). Ao: aorta; PA: pulmonary artery; MPA: main pulmonary artery; RPA: right pulmonary artery; V: probe marker.

Correct positioning of the device was also evaluated during the procedure using transthoracic echocardiography, which also enables the presence or absence of residual flow to be analyzed throughout the device as well as its stability within the duct (Figure 5).

**Figure 6:**
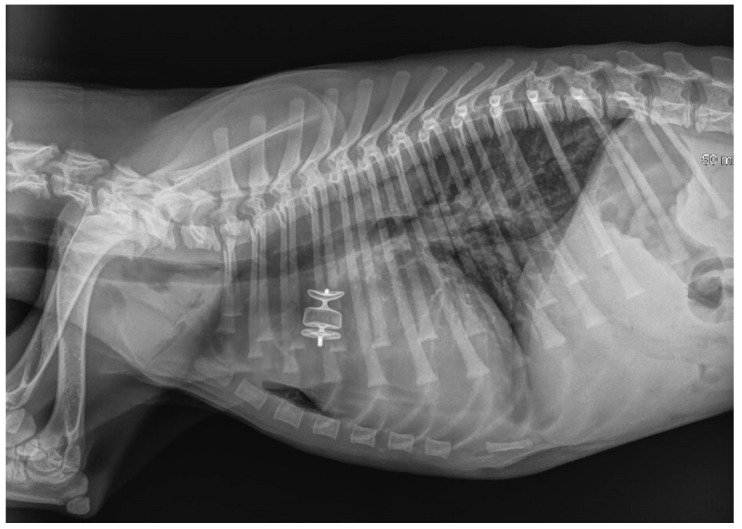
Immediate post-procedure right lateral thoracic radiograph (Case 3). The dog had pulmonary edema before the procedure, which was managed with medical treatment. The device is readily visible in situ. Mild pulmonary infiltrate was still observed in the lung field, especially the caudal lung lobes, after the procedure.

The AVP II device was then detached by counterclockwise rotation of the delivery wire. All catheters were then removed, and the right jugular vein was sutured with 4-0 monofilament polydioxanone absorbable suture (PDSII Ethicon) or ligated [30]. Post-operative thoracic radiography confirmed the correct placement of the device in all patients (Figure 6).

Clinical and echocardiographic check-ups were scheduled one day, one week, and three months after the procedure for all dogs. In all cases with the sutured jugular vein, the integrity of the vessel and flow normality were checked by vascular ultrasound.

## 3. Results

Seven dogs were included in this retrospective study. Three dogs were referred to the Anicura Istituto Veterinario Novara (Cases 1, 2 and 7), and four to the Pavia Veterinary Hospital (Cases 3, 4, 5 and 6) for the noninvasive closure of a previously diagnosed PDA. At clinical presentation, all dogs except Case 3 (which was dyspneic and had pulmonary edema managed with medical treatment before the procedure), were bright, alert, and responsive. All dogs presented a loud, continuous murmur at the left axillary area with a palpable precordial thrill and bounding femoral pulses. Color Doppler echocardiography confirmed the presence of the PDA with left-to-right flow in all the dogs, with a peak transductal flow velocity ≥ 5 m/s.

Case 3, on presentation, was dyspneic with a respiratory rate of 60 breaths per minute with cyanotic mucous membranes. Physical examination revealed pulmonary crackles. Lung ultrasound revealed numerous B lines in all lung fields. Focus cardiac ultrasonography showed severe left atrial and left ventricular enlargement and a restrictive transmitral flow pattern. The dog was managed with an IV constant rate infusion of furosemide and the pulmonary edema resolved in 10 h. When the patient was clinically stable, a complete echocardiogram was performed. There were no complications after the PDA closure and there was a complete resolution of the pulmonary edema. The dog was discharged with torasemide (0.1 mg/kg PO q24 h) and pimobendan (0.3 mg/Kg PO q12 h) for two months. The pharmacological treatment was interrupted one month before the last echocardiographic evaluation.

Table 1 reports clinical data, the devices, and all their technical characteristics (order numbers, diameters and lengths), selected to close the PDA based on transthoracic echocardiography.

**Figure 7:**
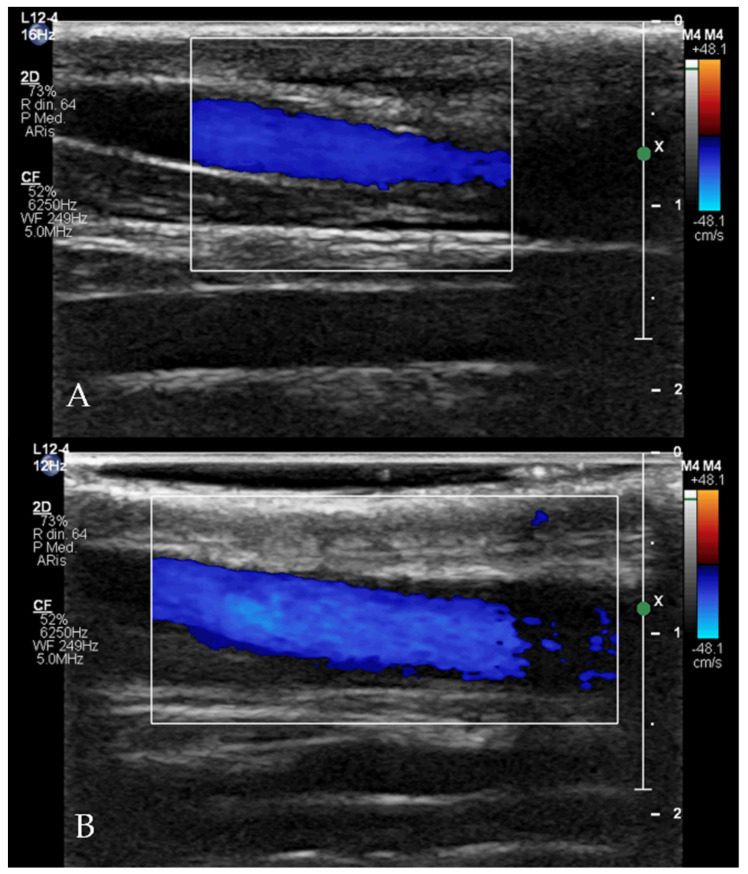
Doppler flow evaluation with linear probe (12 Hz) of the up (**A**) and down (**B**) jugular veins three months after the procedure (Case 5) demonstrating normal size of the right jugular vein compared to the left one, and normal flow within the vessel.

The procedures were all performed as described in the Material and Methods with no complications. The total procedure time was between 20 and 45 min for all patients. After 24 h, the devices were visible within the ductus with no obvious extension into the pulmonary artery and no residual flow across the device in all dogs. The one-week and three-month echocardiographic examinations showed no residual flow across the device in any of the dogs. All patients showed a significant reduction in the left heart size after one week and three months (Table 2). Cases 3, 4, 5 and 6 were subjected to jugular vein suture and flows were normal three months after the procedure (Figure 7).

**Figure 8:**
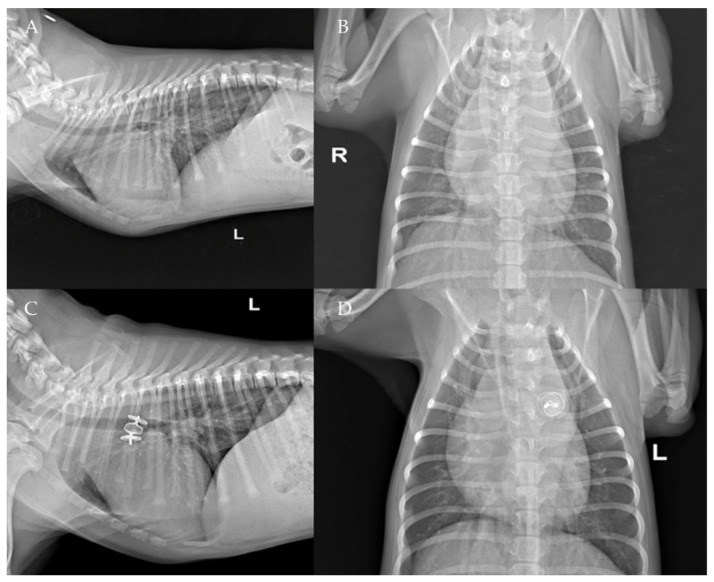
Pre-procedure (**A**,**B**) and post-procedure (**C**,**D**) left lateral and dorso-ventral thoracic radiographs of Case 1. L: left; R: right.

**Figure 9:**
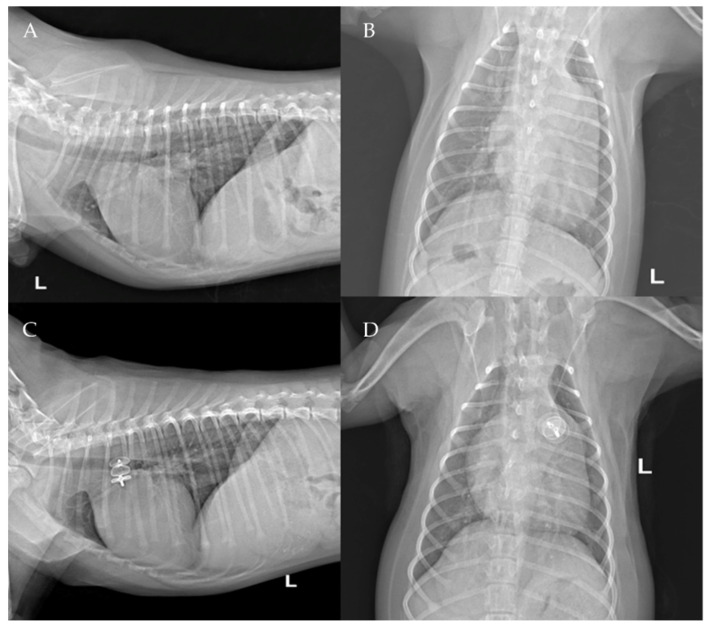
Pre-procedure (**A**,**B**) and post-procedure (**C**,**D**) left lateral and dorso-ventral thoracic radiographs of Case 2. L: left; R: right.

**Figure 10:**
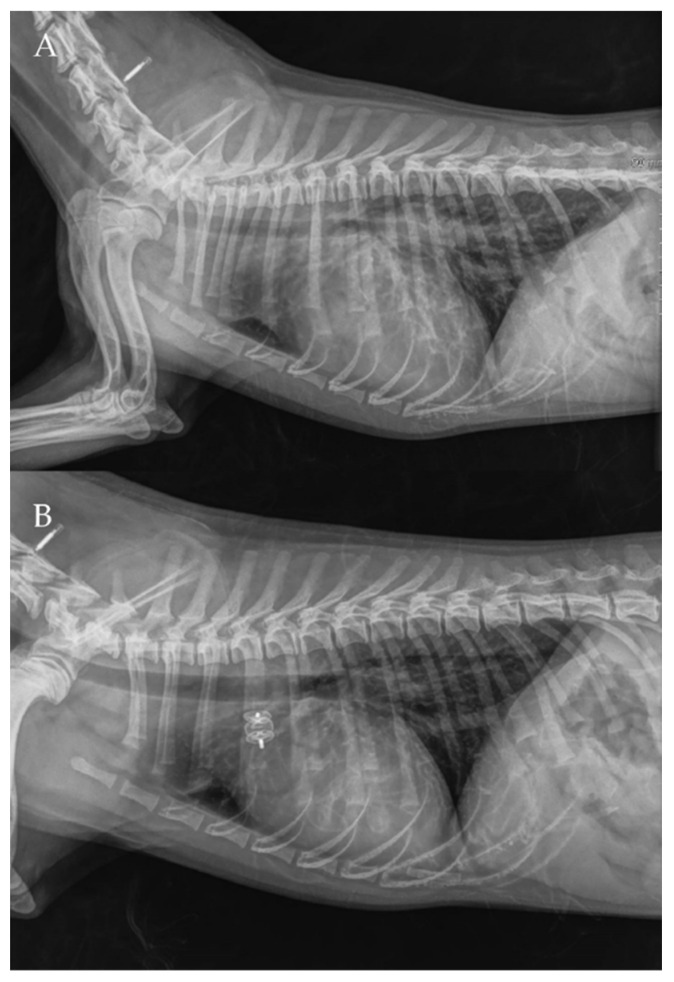
Pre- (**A**) and post-procedure (**B**) right lateral thoracic radiographs of Case 4.

**Figure 11:**
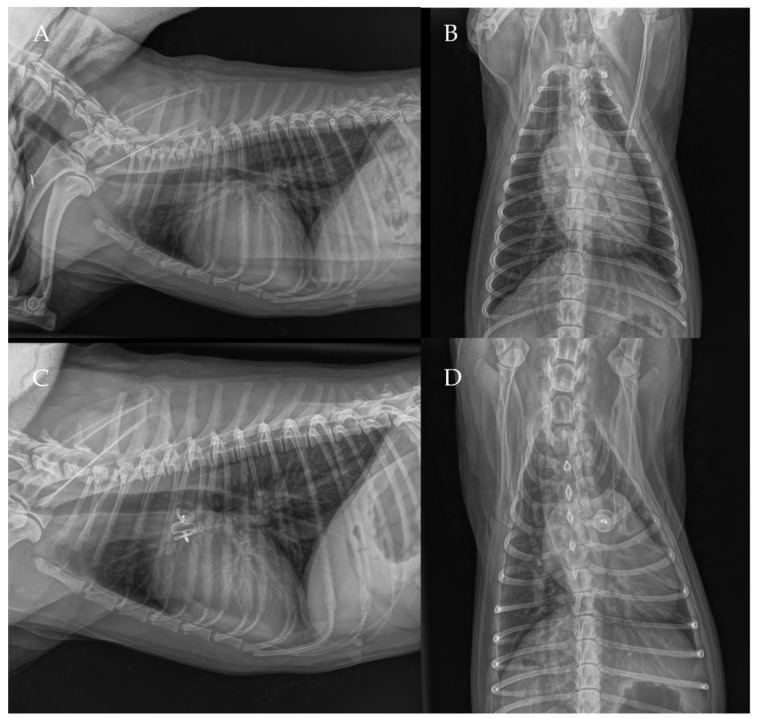
Pre-procedure (**A**,**B**) and post-procedure (**C**,**D**) right lateral and dorso-ventral thoracic radiographs of Case 6.

**Figure 12:**
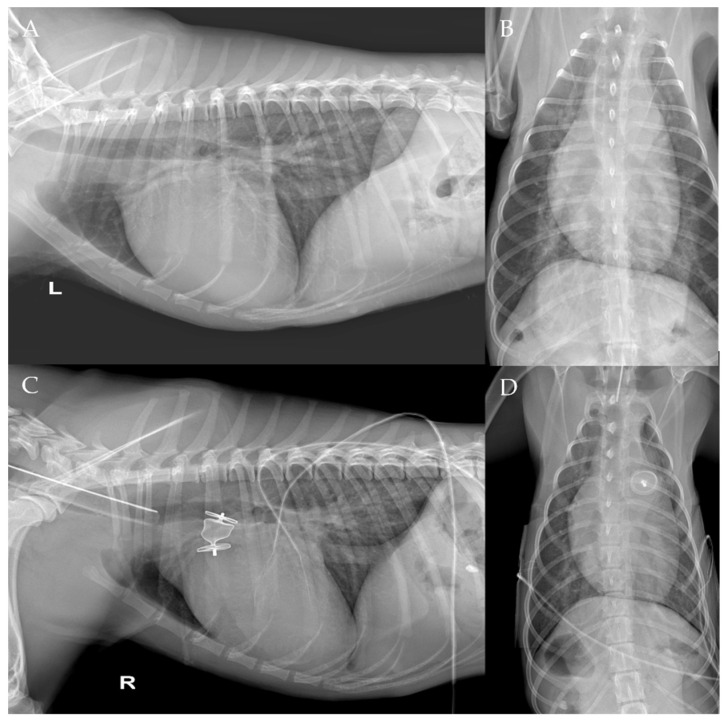
Pre-procedure (**A**,**B**) and post-procedure (**C**,**D**) right and left lateral and dorso-ventral thoracic radiographs of Case 7. L: left; R: right.

The thoracic radiographs performed before and just after the procedure showed a reduction in the cardiac silhouette as well as significant reduction of the over-circulation vascular pattern for all dogs (Figure 8, Figure 9, Figure 10, Figure 11 and Figure 12).

## 4. Discussion

A high mortality rate is reported in dogs that do not undergo PDA closure within the first year after diagnosis [15,31,32,33,34]. For interventional closure of PDA various types of devices have been used in dogs, mainly coils and ACDO using a transfemoral arterial approach [19,32,33]. The main limitations of this transarterial approach are the risk of femoral artery hemorrhage after the procedure [32,33,35], the risk of femoral nerve trauma, and the patient’s size [26].

The present case series describes the use of the AVP II as a first-choice device for PDA occlusion using a transjugular technique in five puppies, of which 4 out of 5 had a body weight of less than 3 kg and 1 out of 5 was a large breed dog, and two adult small-sized dogs (respectively weighing more and less than 5 kg). The AVP II was originally developed for the occlusion of high-flow peripheral vessels such as arteriovenous malformations in human patients [36,37,38,39], but it has also been successfully used for interventional PDA closure in humans and dogs mainly using a transfemoral venous approach [22,23,40,41]. The transjugular approach is widely used in humans and dogs for minimally invasive procedures necessitating right heart catheterization, such as pulmonary balloon valvuloplasty, pacemaker implantation and radiofrequency ablations [25,27,29], however, it has been rarely reported for ACDO release in dogs with PDA [42,43]. Among the venous approaches, the larger size of the vessel and the ease of isolation make the trans-jugular approach more accessible than the femoral one, and this reduces surgical or procedure times [22,26,34,44,45]. Another advantage of the jugular access is that the vein can be sutured and not ligated, as was performed in 4 out of 7 dogs in the present case series. Considering the commonly young age of dogs undergoing PDA occlusion, it can be particularly useful to preserve the right jugular vein for potential future procedures (e.g., central venous access or pacemaker implantation). Lastly, we believe that the shorter distance between the jugular vein and the heart significantly reduces the length of the catheters, which are therefore shorter and more manageable for the surgeon during the procedure.

The AVP II device size selection was based on the type and size of the duct which was at least twice as large as the PDA minimal ductal diameter and at least 20% larger than the PDA maximal ampulla diameter as previously described [22,23]. The literature also reports an oversize factor of 130–150% of the PDA ampulla diameter, as per the manufacturer directions of use [23]. Furthermore, it has been additionally described that in cases that have a PDA ampulla diameter smaller or equal to 10 mm (as in 6 of our 7 cases) lower oversizing factors (110–130%), were successfully used as the distensibility of smaller ampullas is suspected to be minimal [23]. In our case series we used an oversizing factor of at least 120% of the PDA ampulla diameter in accordance with C, D the previous literature.

The present case series suggests that the use of the AVP II via a transjugular approach is a feasible, safe, and effective method for PDA closure in dogs, even in small patients. Embolization of the device did not occur in any case. Complete closure of the PDA and a significant hemodynamic reduction in pulmonary overcirculation and normalization of left heart size were demonstrated in all dogs. In our experience, the Flexor Balkin delivery catheter used in our study has an ideal curvature of the tip (180°), which allows for an easy approach and an adequate movement of the catheter into the right ventricle irrespective of the diameter of the catheter, even in small dogs. In addition, the Flexor Balkin guiding sheath is very handy due to its short length (40 cm), which is a key element for the accurate execution of the procedure.

The present study was not without limitations. Firstly, the retrospective nature of the study did not make it possible to delineate a study design comparing the outcomes between the dogs treated with the AVP II approach and a control group treated with ACDO. A second limit was the lack of homogeneity in the size, weight, and age of the included dogs. Third, the choice of the dimension of the occlusion device was based only on echocardiographic techniques. This may represent a limitation because a double check of the measurements with the angiography has not been performed. However, the device size selection appeared to be adequate in all cases, showing that the echocardiographic measurements obtained by trained operators together with the AVP II device adaptability properties may be the explanation for the high successful rate described in our case series. Finally, the low number of cases and the short period of follow up (3 months) make it impossible to compare the long-term outcomes with those reported in previous literature.

## 5. Conclusions

The present case series suggests that transjugular PDA occlusion using the AVP II is a safe and effective interventional technique in puppies and adult small breed dogs. It can be considered as a fast alternative to the arterial or venous femoral approach using both the ACDO and the AVP II, particularly in those subjects that have very small femoral vessels.

## Figures and Tables

**Table 1:** Clinical data, minimum ductal ostium and ampulla diameters, dimension of Cook Flexor Balkin Guiding Sheath catheters at delivery, and Amplatzer Vascular Plug II order numbers and sizes (pre-implanted device length, minimum internal diameter required and maximum length) for each patient.

Case	Breed	Sex	Age	Weight (kg)	PDA Ostium (mm)	PDA Ampulla (mm)	Cook Flexor Balkin Guiding Sheath (Fr)	AMPLATZER Vascular Plug II Order Number	AMPLATZER Vascular Plug II Diameter (mm)	Pre-Implanted Device Length (mm)	Minimum Internal Diameter Required (inches)	Maximum Length (cm)
Case 1	Poodle	M	4 months	2.8	2	6	6	9-AVP2-010	10	7	0.070	100
Case 2	Poodle	F	4 months	2.9	3	7	6	9-AVP2-010	10	7	0.070	100
Case 3	Border collie	F	3 months	2.9	2.7	10	6	9-AVP2-012	12	9	0.070	100
Case 4	Maltese	M	6 months	2.9	2.4	6.6	5.5	9-AVP2-010	10	7	0.070	100
Case 5	American Staffordshire Terrier	F	3 months	8.5	2.8	8.6	6	9-AVP2-012	12	9	0.070	100
Case 6	Poodle	F	5 years	7.3	2.9	9.1	6	9-AVP2-012	12	9	0.070	100
Case 7	Poodle	F	1 year	4.6	3.4	11.2	7	9-AVP2-014	14	10	0.086	100

**Table 2:** Echocardiographic measurements before transvenous Amplatzer Vascular Plug II occlusion (T0) and at 24 h (T1), one week (T2), and three-month check-ups (T3).

Case	Timing	LA/Ao	LVIDDn
Case 1	T0	1.55	2.02
	T1	1.41	1.80
	T2	1.30	1.60
	T3	1.20	1.47
Case 2	T0	1.68	2.16
	T1	1.32	1.48
	T2	1.32	1.37
	T3	1.30	1.30
Case 3	T0	1.85	2.96
	T1	1.41	2.16
	T2	1.10	1.56
	T3	1.10	1.64
Case 4	T0	1.39	2.52
	T1	1.26	1.84
	T2	1.24	1.75
	T3	1.21	1.60
Case 5	T0	1.22	1.84
	T1	1.23	1.68
	T2	1.19	1.69
	T3	1.20	1.69
Case 6	T0	1.33	2.46
	T1	1.18	1.73
	T2	1.10	1.65
	T3	1.10	1.52
Case 7	T0	1.90	2.20
	T1	1.55	1.90
	T2	1.39	1.50

T0, pre-operative echocardiographic evaluation; T1, 24 h post-operative echocardiographic evaluation; T2, one-week echocardiographic check-up; T3, three-month echocardiographic check-up; LA/AO, left atrium to aorta ratio; LVIDDn, left ventricular internal diameter in diastole normalized for body weight.

## Data Availability

The data presented in this study have not been published elsewhere but are available on request from the corresponding author.
